# Regarding the Pain of Others? Contradictions Between Public Discourse and the Lived Experience of Pain

**DOI:** 10.3390/healthcare12232466

**Published:** 2024-12-06

**Authors:** José A. Cerrillo-Vidal, Mª Isabel García-Rodríguez, Rafael Serrano-del-Rosal

**Affiliations:** 1 Department of Sociology, Social Sciences Faculty, Universidad Pablo de Olavide, 41013 Seville, Spain; jacervid@upo.es; 2 Institute for Advanced Social Studies, Spanish National Research Council (IESA-CSIC), 14004 Córdoba, Spain; igarcia@iesa.csic.es

**Keywords:** pain management, social perceptions of health, qualitative research, legitimacy, attitudinal fallacy

## Abstract

Background/Objectives: Pain is a complex and subjective experience influenced by psychological, cultural, and social factors. This study aims to investigate how social perceptions of pain affect the lived experiences and coping mechanisms of individuals suffering from pain. By comparing public discourse with the experiences of sufferers, we explore whether the social legitimacy of pain influences how it is managed. Methods: A multi-phase qualitative study was conducted, comprising eight focus groups with members of the Spanish general population and 19 in-depth interviews with individuals suffering from various types of pain. The focus groups explored social perceptions of pain’s legitimacy, while the interviews delved into the sufferers’ personal experiences. The data were analyzed using thematic analysis to identify patterns and disparities between public discourse and individual narratives. Results: The focus groups revealed widespread social empathy towards all types of pain. However, interviewees reported significant social pressure to normalize their behavior and downplay their pain. Many felt misunderstood, unsupported, and stigmatized, especially in environments where they held subordinate roles, such as the workplace or healthcare settings. Sufferers often resorted to silence or isolation to avoid judgment. Conclusions: While Spanish society outwardly legitimizes all forms of pain, sufferers experience significant discrepancies between public empathy and actual social support. The findings suggest that raising awareness alone is insufficient, and that structural changes are needed to address the daily burdens that individuals face when coping with pain, particularly in workplace and healthcare environments.

## 1. Introduction

The International Association for the Study of Pain defines pain as “an unpleasant sensory and emotional experience associated with, or resembling that associated with, actual or potential tissue damage.” This definition, slightly modified in 2020 [1] (, continues to emphasize the experiential nature of pain, as it has since 1979. Although pain often has a physical (nociceptive) component, it is the brain’s interpretation of certain stimuli that allows us to perceive it as pain. In other words, a person may experience an unpleasant sensation, regardless of its origin, which may not necessarily be classified as pain. It is only when the cognitive system associates the stimulus with pain that it is recognized as such. Conversely, the brain may signal the presence of pain without an apparent or identifiable source. Pain is, therefore, a fundamentally internal and subjective experience, making it difficult to objectify or measure, and often hard for others to comprehend.

Since pain is mediated by the brain, it is expected to be influenced by social, cultural, psychological, and other factors that shape our consciousness. As the social communication of pain model shows [2], biological, psychological, and social factors intervene in complex ways in the expression and management of pain in all of its phases. Individuals are, due to their trajectory and socialization, predisposed to adopt a series of responses to pain, even before the events that trigger it occur. In turn, culture influences how pain is perceived by others, which leads them to adopt different actions that can contribute to alleviating, maintaining, or even intensifying such pain. Indeed, it is well-documented that the experience of pain has evolved over time, with each culture adopting unique bodily practices (hygienic, dietary, sexual, etc.) and forms of pain expression that impact how pain is experienced [3,4]. Studies also show that pain experiences and coping mechanisms are shaped by factors such as age [5], socioeconomic status, [6,7] gender (Bartley and Palit, 2016; Pieretti et al., 2016) [8,9], family environment [10,11], and ethnicity or race [12,13], among others [14,15].

To understand pain and develop effective management strategies, it is crucial to study the social context of those who suffer from it. Across human societies, certain types of pain, or the pain experienced by some collectives, are considered more deserving of attention than others. For instance, chronic pain, despite being known since ancient times, was practically considered a mental disorder until the 1970s [3,4,5,6,7,8,9,10,11,12,13,14,15,16]. Also, research shows that women and ethnic minorities receive fewer painkillers despite reporting higher levels of pain than men or white patients (Dusenbery, 2019; Ezenwa, 2012: McGregor, 2020; Rambachan et al., 2023) [17,18,19,20]. Different meanings or functions are assigned to various types of pain, and sufferers must conform to socially accepted verbal and physical expressions to make their pain understandable and elicit the desired response from others [3,4]. These factors significantly impact how pain is experienced.

Given this, we must ask how social perceptions influence the individual experience of pain. In this paper, we first explore Spanish society’s perceptions of pain based on focus group data. We examine interviews with individuals suffering from various types of pain to assess how social perceptions, as discussed in the focus groups, affect their feelings and coping mechanisms. Although collective narratives suggest that contemporary society views all types of pain as legitimate and believes that care should be provided regardless of the pain’s nature, those experiencing pain lament the lack of understanding and empathy from others, including those closest to them. The possible causes of these disparities and their implications for understanding and managing pain are discussed.

## 2. Materials and Methods

Since pain is an internal sensation that heavily depends on the sufferer’s experience and subjectivity, it is particularly challenging to study. Pain does not lend itself to the standardization needed to quantify variables, as demonstrated by repeated failures to create questionnaires that can objectively measure it [21]. It is also true that pain is difficult to approach using qualitative techniques, as it is a sensation that can be hard to verbalize. However, the fundamentally subjective nature of pain makes qualitative methodology the most appropriate for its study.

With this in mind, we have designed a multi-phase study to explore Spanish society’s perceptions of the legitimacy of pain. We sought to distinguish between the perspectives of those suffering from pain and those of society at large. Our goal was to determine if there are differences in how pain is understood and managed by those who experience it compared to the predominant pain narratives and stereotypes circulating in Spanish society. To achieve this, we used two different research techniques, as follows: open-ended interviews of people who actually suffer from pain and focus groups for the collective discourse on pain. The interview method has the ability to record the expressive functions of language and uncover narratives beyond the most stereotyped collective discourse [22], making it the most appropriate method to capture the experience of people suffering from pain. Social perceptions, on the other hand, were explored through focus groups. While the focus groups shared the same qualitative logic as the interviews, their discussions had a markedly lower subjective and expressive component. In focus groups, participants are encouraged to find common ground, which often leads to the articulation of familiar, stereotypical, or institutionalized narratives [23].

Starting with the focus groups, eight groups, each composed of nine participants, were designed. The variables considered were gender, age, and employment status. For employment, we took into account job stability, sector (public or private), position within the company (owner, employee, etc.), and, most importantly, whether the job required continuous physical exertion. All groups were located in urban environments, in cities with at least 300,000 inhabitants, which is where the vast majority of the Spanish population resides, with the exception of group 8, which was located in a small rural town precisely because we were looking for more traditional discourse. However, these variables were not systematically cross-checked to generate a pre-planned discussion map. Instead, we mixed different dimensions to form groups of relatively homogeneous composition, which were still heterogeneous enough for relevant variations to emerge. While it would have been possible to include additional groups representing broader social collectives, despite important differences, the discussions remained remarkably consistent on some key points, as will be seen. We can, therefore, state that a reasonable degree of thematic saturation was reached [24], sufficient to meet the study’s objectives. The complete sample is shown in Table 1.

An outline script was developed and adapted to each group’s particularities. The script was organized around four main topics, as follows: (1) a general discussion on pain, its intensity, and how to manage it (*“how intense is the pain you’re feeling?” “How do you cope with it?”*); (2) identifying differences between types of pain (*“Is this kind of pain different from this other one?” “What are the differences between them?”*); (3) discussing the legitimacy of various types of pain (*“Is this kind of pain more or less worthy of being attended than others?” “Why?”*); and (4) a final section featuring more specific questions about aspects such as pain’s relationship with other areas of life (mostly, work, leisure, and family), individual responsibility for pain, and associated stereotypes. As is customary in this research method, the moderator let the participants raise topics of interest, intervening only when the group did not spontaneously arrive at the topic. These interventions were made using a hands-off approach, building on the participants’ prior statements. The topic used to initiate the discussion was the group’s perception of their general health.

Participant recruitment was conducted with the assistance of a specialized company. As is typical in qualitative research, the participants were given a small token of appreciation as an incentive to attend and actively participate in the discussions. The fieldwork took place from mid-January to mid-February 2019.

The interview sample, on the other hand, was purposive, with the primary criterion being the inclusion of a diverse range of pain sources, i.e., the origin of the pain, regardless of other types of secondary pain. For example, severe physical pain may lead to depression, while psychological or social pain, such as that caused by addiction or bullying, can manifest as physical pain. We aimed to identify commonalities and differences in the narratives of sufferers depending on the source of their pain. The sample was organized by grouping the different types of pain into four broad categories, as follows: (1) physical pain of purely nociceptive or organic origin; (2) psychological pain associated with psychiatric diagnoses; (3) emotional pain, understood as the “internal response to noxious psychological stimuli” [25]; and (4) social pain, defined as “an unpleasant emotional experience triggered by feelings of exclusion or rejection from people or social groups with whom one wishes to establish a relationship” [26]. This classification has been successfully used in previous quantitative and qualitative studies that inspired the current project [27,28]. However, this classification is only indicative and not exhaustive, as certain types of pain may fall into more than one of the four categories, or somewhere in between. The complete sample is shown in Table 2. 

The recruitment of informants was carried out through both the services of a qualitative fieldwork company and collaborations with associations and institutions specializing in the treatment of various diseases and social problems. Children under 18 years of age were excluded from the sample.

While the interview script contained a series of core questions and topics of interest, it was flexibly adapted to each interviewee to accurately capture their unique characteristics and specific communicative situations. The script was organized around four main thematic areas, as follows: (1) individual factors, focusing on the subjective experience of pain (e.g., *“Has the pain affected your mood?” “Has it changed your perception of life in general?” “How responsible do you think you are for the appearance of the pain you suffer from?”*); (2) structural aspects, examining how pain affects various areas of the participant’s life (e.g., *“Has the pain affected your employment?” “Have your relationships with family and/or friends changed?”*); (3) social policies, focusing on the relationship between pain and social welfare systems, particularly healthcare (e.g., “*How has your experience in the healthcare system been?” “Has it been easy for you to request sick leave if you needed to?*”); and (4) quality of life, analyzing the impact of pain on daily activities and different coping strategies (e.g., *“Have you needed support to cope with pain?” “What do you do to deal with pain in your daily life?”*). Additional impromptu questions were also included as needed during the course of each interview.

The fieldwork was conducted between November 2019 and April 2020. Most interviews were conducted face-to-face, however, due to the COVID-19 pandemic, some of the later interviews had to be carried out via online communication platforms. There were no significant differences in the quality or depth of interactions between face-to-face and virtual interviews.

The project was approved by the Cordoba Research Ethics Committee on January 28, 2020 (Minute No. 299). Both interviewees and focus group participants received an information sheet and signed informed consent forms prior to participating.

The interviews and focus groups were digitally audio-recorded and transcribed following standard conventions for distinguishing participants, silences, tone of voice, and overlapping speech.

The transcripts were thoroughly analyzed using Atlas.ti V9.0 and Atlas.ti web software V5.0. We followed [29] three-level analysis model, which combines various textual and formal analysis techniques with contextual and thematic analysis. This method was used to relate the results to broader socio-historical dimensions, particularly in relation to previous studies conducted by our research group.

## 3. Results

As previously mentioned, the main objective of this paper is to contrast public discourse on the legitimacy of pain, as gathered from focus groups, with the narratives of individuals suffering from pain of different origins, based on personal interviews. Consequently, the results are divided into two distinct subsections.

### 3.1. Social Perceptions of Pain

The focus groups revealed that perceptions of pain vary across social groups depending on their life circumstances. For instance, the younger individuals tended to focus almost exclusively on emotional pain, which is logical considering their overall good health and limited experience with the gradual bodily decline associated with aging. In contrast, women nearing retirement age, particularly those in physically demanding jobs, such as cleaners or nurses, frequently discussed physical pain but often intertwined it with concerns about care, as follows: “Who will take care of me now that I am aging? I have cared for my husband and children, and now they don’t want to care for me.” This pattern was also present in other groups, with pain being tied to concerns specific to each group’s age, gender, and employment status.

Despite these differences, common themes emerged across all groups, which can be considered prevalent discursive topics in Spanish society. The first is the strong connection between the somatic and the psychological or emotional states, which are clearly expressed in pain. Emotional states influence bodily processes, manifesting unresolved or poorly managed stress, anxiety, or depression as physical pain. The emotional state also affects the intensity and characteristics of physical pain, which can be observed in the following example:

Woman (W from now on): “Well, I had a hard time understanding that it was really like that. I mean with me... it all started with digestive problems and so on... and as far as I was concerned, that was totally separate from... what was going on in my head, which was terrible anxiety, depression and so on and. And it was really hard because I also have a scientific background and I needed to understand that link. And it took me a lot to say, ‘I was kind of manifesting everything in here physically, right?’ and understand the mechanisms, and now we’re starting to see what this physiological connection is like. And it’s there, but I think it’s what we see now, maybe, more common sense and we see it here maybe by chance, we don’t tend to make that connection often”.

**G7**

The relationship between physical and emotional pain can also work in the opposite direction, as follows: a physical problem can lead to depression and other psychological distress, particularly in cases of chronic- and severe-pain-causing illnesses, such as fibromyalgia, which can be observed in the following example:

W: “I think that long-term aches and pains are the ones that affect you the most psychologically because you realize that they aren’t getting better, that they aren’t going away, and you realize that you are always in bad shape. I think that those pains are the ones that... What I’m saying is that these girls that have the, that fibro thing, they’re really really... and, to be honest, they’re on antidepressants because of how bad they’re coping with the, with the pain bit”.

**G4**

The interconnections between the somatic, emotional, and psychological aspects of pain led to a second key point of consensus across all of the focus groups, as follows: a strong legitimization of all types of pain, regardless of its source or cause. Psychological, emotional, or social pain were considered to be just as legitimate as physical pain, and these were often compared. The participants stated that if one sees a healthcare professional to treat physical pain, the same should be carried out for psychological or emotional pain, as follows:

Man (M from now on): “And I asked for help at home and whatnot and my family’s response was: ‘Well, let’s see, if you had toothache, we’d send you to the dentist, so if you’re in a bad way, whatever your problem is, if you really think, I’m mean you’re old enough, and you think you should see a psychologist, then it’s just like breaking your leg and you need to pay for whatever it is.’ Sure, I really appreciated that answer and I’ve been telling people about it a lot”.

**G6**

The legitimization of psychological and emotional pain emerged even among those who might have been expected to show resistance to it, such as older men from rural backgrounds with physical jobs (agriculture, fishing, and construction). Despite their strong traditional masculine identities, which could have led them to dismiss emotional or psychological pain as a character flaw [30], these men also viewed psychological pain as being just as harmful as physical pain. The same can be said for the group of business owners and self-employed participants, who might have been skeptical of emotional or psychological pain as potential excuses for absenteeism. Yet again, this group equated psychological pain with physical pain, such as in the following example:

M: “And the people around you, if you don’t have a head injury or a broken leg… I wanted to touch on that too. Not just physical pain, but also psychological pain. There’s this invisible pain, and that’s mental pain. Totally misunderstood, nobody gets it, because unless you’ve got a head injury or a broken leg… ‘Go for a walk’ (…) they say”.

M: “And it’s crippling, that pain”.

W: “‘Go for a walk, go out, have fun’ and ‘this is all just nonsense, go for a walk’ and no. It’s a disease like any other that must be treated. It’s a… let’s say, a chemical imbalance”.

**G7**

As we can see, there was broad acceptance that psychological and emotional pain is as legitimate as physical pain, and it is reasonable to say that this notion is firmly established in societal discourse in Spain. This was one of the most surprising and unexpected findings of this research. Overall, the focus groups indicated that Spanish society views pain as an obstacle to personal fulfillment and quality of life, which are often synonymous with health. Moreover, it grants a high level of legitimacy to all types of pain, regardless of origin, and recognizes deep interconnections between the somatic, emotional, and psychological dimensions. These findings suggest that Spanish society demonstrates significant empathy toward all forms of pain.

### 3.2. Perceptions of People Suffering from Pain

In contrast, the interviews presented a starkly different picture. An overwhelming majority of pain sufferers expressed feeling misunderstood and noted a lack of sensitivity from those around them. This lack of understanding is often reflected in intense pressure to return to normal life, as follows: sufferers are pushed to resume work as soon as possible (and at the same level of competence and productivity), or to return to family obligations, school, or shared leisure activities. In short, they are expected to “get back to normal” as soon as possible, which can be observed in the following example:

W: “Now, what I haven’t got, where I haven’t had support, has been from people, for example, who are not familiar with this pain, for example, when I have been working, sometimes from my bosses… From my managers. They didn’t believe it. Especially at first, they thought that I was something that, I was doing it either to get out of going to work or that I was exaggerating my… my pain”.

**I2**

When sufferers are unable or reluctant to meet the expectations placed upon them, those around them often begin to question the legitimacy of their pain. This starts by doubting the intensity of the pain the individual claims to feel, assuming that they are exaggerating or lack the willpower to cope with the problem. In some cases, the existence of pain itself is questioned, and sufferers are accused of faking it for attention or for personal gain. Alternatively, their pain may be attributed to personal failings, such as laziness, weakness, or a lack of self-control. Particularly for conditions with less social legitimacy or those harder to visualize or understand, the existence of the condition itself may be doubted. In such cases, either the sufferer or the cause of their pain—or both—are delegitimized.

W: “I have friends who don’t get it, I have friends who think I’m a wimp and they’re friends and they love me, but they don’t quite get it (…) Tell me you don’t want to hear about it, that you’re fed up, that it’s no big deal, I don’t know, that would work better for me than telling me… like saying: you don’t want to be the center of attention. Because that really hurts, you know, because it’s like, you ask me how I’m doing, then I tell you and you don’t want to hear about it. Don’t tell me how I’m doing”!

**I9**

In this scenario, the sufferer must demonstrate, either through objective evidence (such as expert reports) or through gestures and behaviors, that they are indeed in pain and struggling to perform their daily tasks. People in pain are, therefore, constantly judged and continually forced to justify their condition, such as in the following example:

W: “(…) That’s what’s hardest for me… much more than what I’m going through. I deal with it, but it’s the ignorance and constant judgment… it’s like, ask me, you know? Ask me, let’s talk. If I tell you, don’t judge me beforehand. I have no plans to stop working… it doesn’t interest me at all; my work is my life”.

**I4**

Any deviation from behavior deemed acceptable for someone in debilitating pain may prompt immediate questioning of their condition. Interestingly, such pressure can even come from people with the same condition, as follows:

M: “(…) Once they made a… a pharmacist; they made a calendar of people with osteoporosis. They picked [people] and… it turns out that later someone from the association, when they saw the calendar, said, ‘I don’t know…’ They said, ‘You can’t tell she has osteoporosis,’ and I said, ‘Of course, because if they make you up, put on a nice dress, do your hair and makeup, and take a nice picture of you, it looks amazing’… But, of course, your bones are on the inside”.

**I1**

Sometimes, even providing objective evidence is not enough, and the sufferer is asked to ignore their pain and return to the normal rhythm of everyday life. On other occasions, they are advised to seek quick fixes to normalize their behavior, even if this does not alleviate the pain or address its root cause, such as taking drugs for instance, or the suggestion to forget the cause of the pain and what it has meant in the life of the person with the pain in order to not expose it to the social environment and avoid treatments. It is about distancing the person with pain from social judgement and stigma, in the belief that this action can avoid the pain. For instance, the mother of a girl who had suffered bullying told us how some therapists recommended her not to report her daughter’s bullies, as follows:

M: “(…) So we called the psychiatrist, we called the psychologist, and the psychiatrist, who is also a friend of the doctor I’m working with, says to me: ‘Is your daughter happy now?’ or ‘Is she any happier than before?’ He says, ‘Go away,’ he says, ‘Don’t keep doing this,’ he says, ‘Don’t continue, because it’s going to take another year until… she’ll have to go through a lot. I’m telling you as a father and as a forensic psychiatrist, they’re going to put her through psychiatric tests. This city [anonymized toponym] is very small, and she will come across them, and I don’t think it’s worth it unless you want the money’”.

**I18**

As can be seen, the sufferer’s condition becomes more of a stigma than a normalized state, as [31] describes, as follows: an attribute that diminishes the value of interactions for those who carry it, making them less desirable in the eyes of others and leading it to become the predominant identity trait of those who suffer from it. The interviews suggest that there may be differences in the degree of legitimacy afforded to certain ailments, with a more pronounced effect for pain caused by conditions or illnesses with little or no social legitimacy. These include obesity, addictions, and Multiple Chemical Sensitivity, as well as conditions that, although medically recognized, have less obvious causes, less visible consequences, or are difficult for the general population to understand, such as fibromyalgia, lupus, and depression. Nevertheless, as some of the quotes above demonstrate, pressure on sufferers is widespread and can be felt even in highly legitimized circumstances, such as the loss of a loved one.

The same can be said of the different spheres in which sufferers operate. There appears to be less understanding of pain and greater coercion to return to normality in situations where sufferers are in a subordinate or dependent relationship. This clearly includes the workplace, as well as interactions with healthcare professionals and other public and private expert systems, such as in the following example:

W: “Yes, yes, yes, of course, I’m not saying I trashed a consultation room because he didn’t believe I was sick, and I said to him, ‘Can’t you see me? Can’t you see that I’m losing my mind?’ And I was so lost, I, I, I smashed the place up, like trashed it, because… because they don’t believe you. I’m telling you what’s happening to me, and you’re the doctor, and you tell me that I just want to be signed off? No, no, I don’t want to be signed off—I get paid less when I’m signed off, asshole”!

**I8**

Some interviewees excused the systematic mistrust from expert systems toward sufferers as a way to prevent fraud or abuse in the granting of benefits or leave. Other times, they attributed it to a lack of experience with specific conditions, such as in the case of general practitioners dealing with rare illnesses or psychological disorders. However, this distrust of the sufferer, the questioning of their condition, and the pressure to resume daily activities also occurred within primary groups. While many interviewees identified support from family and friends as essential in helping them to cope with their condition, it sometimes required educating others to help them to overcome doubts and understand the limitations faced by the sufferer. In some cases, however, primary groups were equally unsympathetic, just as skeptical, and might even write off the sufferer as incapable of fulfilling their socially assigned roles, such as in the following example:

M: “There’s another family there, from one of the dads in the group, who rally around more, and I’ve got a family that doesn’t… They don’t help at all. At the beginning, it hurts, but eventually, you get used to it… Well, you find that there are other parents with the same story, where the family disappeared. In fact, one mother there, her sister lives in the same block, and she hasn’t been to see her. Her son died three years ago, three years ago this Christmas, and in all that time, she hasn’t been to see her, right? That’s how it is. So, I’m telling you, it’s not just about friends, it’s also about family”.

**I11**

In fact, some interviewees saw the support they received from those close to them as a way to gauge the strength of their bonds, as follows: those who showed support and affection versus those who did not understand their situation and chose to break ties. Again, this seems to especially affect less socially legitimate forms of pain but has also been reported by interviewees who had experienced highly legitimate pain, such as bereavement, betrayal by a partner, or gender-based violence. It even occurs with medically recognized pain, such as lupus. In short, the same delegitimization processes observed in more institutional or hierarchical settings can also be found within primary groups.

In response to this lack of understanding, sufferers resort to a variety of strategies. Some exaggerate their ailments or feign visible symptoms to make the pain or disability more obvious to others. For example, one interviewee with chronic fatigue syndrome admitted to faking a limp so his friends would realize he could no longer socialize as he used to. Of course, such behavior carries the risk of being discovered, which could further delegitimize their pain. This may explain why many interviewees preferred self-isolation, withdrawing from collective activities or cutting the weakest ties in order to avoid constant questioning and reduce the likelihood of being unfairly judged or misunderstood. As a result, they limit their interactions to those that are most comfortable or least stressful. Most often, however, sufferers opt for silence or concealment; moreover, they refrain from sharing their pain or struggles with others. In doing so, they avoid judgment or questioning and distance themselves from the stigma associated with suffering, such as in the following example:

W: “Now I’ve been trying to hang in there. For me, the cold is awful, and I’ve been stuck outside, freezing to death. And if I say something, it’s like, ‘Look at her, pulling strings, trying to get attention, or whatever.’ So, you put up with it and stay quiet. But of course, you pay for it, because when people are enjoying themselves after a week of work, I’m shut in at home for two days… shut in with pain”.

**I5**

It is clear that concealing the full extent of their ailments comes at a high personal cost, as follows: more pain, higher doses of medication, and time off spent recovering from overexertion. The long-term adverse effects of these behaviors can accumulate and lead to serious physical or psychological harm. However, it seems these costs are preferable to the constant pressure from their environment. One can only imagine the magnitude of that pressure if sufferers feel compelled to endure pain in silence to escape it, with all of the short-, medium-, and long-term personal damage that such a choice entails.

Furthermore, this silent suffering has the effect of *individualizing* the pain. If sufferers do not expect their environment to legitimize their pain—i.e., to understand it and take collective responsibility for its effects—the pain remains a strictly private matter for which the sufferer alone is responsible. This is clearly a Pygmalion effect, as follows: by choosing silence over stigmatization, sufferers confirm to others that their pain is largely their own responsibility, and any visible manifestation of its effects can be seen as a strategy to conceal some form of self-interest or weakness. In other words, by enduring the pain, sufferers reinforce the belief that they can bear it, and perhaps more than they claim. Moreover, the prejudices associated with pain and sufferers, along with the delegitimization strategies and the pressure to reintegrate them into social normality, are somehow validated. Individualizing pain may also divert attention from its root causes—whether biosanitary, psychological, biographical, or social—and shift the ultimate responsibility onto the individual. Finally, the physical, emotional, and psychological costs incurred by the sufferer as they forgo external understanding and care—i.e., full social legitimization of their ailments—become invisible.

## 4. Discussion

The interviews and focus groups presented starkly different perspectives on pain, as expected, given the study’s design aimed at comparing the views of sufferers and society at large using different research methods. It was anticipated that the data obtained from different methodologies would offer differentiated nuances. However, what was unexpected was the extent to which the accounts of sufferers not only differed but, in some cases, directly contradicted those of the focus groups. Based on focus group discussions, it appears that Spanish society has legitimized all types of pain and expresses empathy for those suffering. This might lead us to believe that society is also sensitive to the need to take responsibility for this pain, caring for and protecting those who suffer. Yet, in the interviews, those affected not only felt neglected and unprotected, but often expressed frustration at society’s failure, including family and friends, to understand and empathize with their condition. What accounts for such a significant discrepancy between socially dominant narratives and the perceptions of those in pain? More broadly, what do these differences imply for the study and management of pain?

As mentioned earlier, pain is a deeply internal and subjective experience, making it difficult for others to comprehend its debilitating effects on those suffering. As a result, sufferers are often expected to adjust their behavior and expressions to align with culturally accepted ways of conveying pain. Any deviation from these norms may lead to doubts about the legitimacy of their condition. However, the medical community and other expert systems, which monopolize the legitimate discourse around the body and health in contemporary societies, have failed to establish reliable, objective, and measurable indicators or standards for understanding pain. Could it be, therefore, that, while contemporary society aspires to be sensitive to the pain of others—as suggested by the focus groups—it lacks the cultural tools to make the pain of others intelligible?

Many sufferers are acutely aware of how complex it is for others to understand their pain, as reflected in several of our interviews. Nevertheless, acknowledging this and recognizing that society has not yet developed reliable protocols for understanding pain does not entirely explain the significant disparity between collective discourse and the everyday reality of those in pain. After all, one does not need to have experienced a problem, discomfort, or hardship to sympathize with those who have. Furthermore, being unable to fully understand another’s pain does not necessarily imply mistrust or a lack of compassion, particularly when public discourse claims that all types of pain are legitimate and deserving of care. Something else must explain these discrepancies.

This inconsistency between publicly expressed attitudes and private behaviors—the so-called “attitudinal fallacy”—has long been recognized in the social sciences ([32,33] and has sparked important methodological debates in the last decade [34]. Social pressure often leads us to publicly support socially desirable positions, even if, in private, we behave in ways that openly contradict them, whether we are aware of this inconsistency or not [35] Research methods like surveys or focus groups can activate this bias, as participants may respond in ways they believe are expected or socially acceptable.

However, this does not necessarily mean that certain behaviors or attitudes are more genuine than others or reflect the subjects’ true preferences. Different interaction contexts activate distinct interpretive frameworks and, consequently, lead to different behavioral patterns. As we have seen, when in a group setting, individuals tend to express views that they perceive as socially desirable, such as considering all types of pain to be legitimate. But why do we then deny this same legitimacy when confronted with others who are visibly suffering? It is important to recall that, like illness and death, pain poses *a threat to societal normality*. Pain prevents sufferers from performing their socially assigned roles in the usual way (whether at work, in family or school settings, or even in shared leisure activities). Depending on its intensity, pain can alter sufferers’ perceptions of their own bodies and identities, causing others to view them differently [36]. These changes may provoke a conservative reaction from the social environment, prompting efforts to restore normality by pressuring the sufferer to resume their roles.

This pressure can manifest as doubts about the sufferer’s credibility or the encouragement to quickly reduce their pain to tolerable levels or resolve its causes (e.g., through medication or “miracle” therapies).

At this point, it is worth reexamining whether public discourse and private behaviors regarding pain are truly so incompatible. As it turns out, the focus group participants also expressed doubts about individual responsibility for one’s own pain. This is related to one of the core concepts of health and pain that was discussed across all groups, being the intense relationship between the physical, psychological, and emotional states. Indeed, if physical and psychological pain are so intertwined, could changing one’s attitude or emotional state be sufficient to stop experiencing physical pain, which is often the manifestation of psychological discomfort? This topic was widely debated in the focus groups, and although the participants seldom reached universally satisfactory conclusions, many argued that individuals are ultimately responsible for their own health. This belief in the interconnectedness of physical, psychological, and emotional pain could help to justify the societal pressure on sufferers to return to normality, thus reinforcing the individualization of pain to which they are often subjected. Such an approach would also help to reconcile the contradictions between the empathetic rhetoric toward sufferers and the practical pressures they face in everyday life.

## 5. Conclusions

Our research findings highlight a significant contradiction between public discourse and everyday behaviors concerning the legitimacy of pain. Publicly, all types of pain are declared legitimate, and there seems to be a high level of empathy and understanding for those suffering from pain. However, the testimonies of individuals actually suffering from pain reveal a very different reality. There is a pervasive tendency to delegitimize others’ pain, questioning their credibility and pressuring them to return quickly to their normal roles, regardless of whether their pain persists or its causes remain unresolved.

This discrepancy can partly be explained by the difficulty in comprehending others’ pain, a fundamentally internal and subjective experience. This is particularly true in societies like ours, where there are no clear cultural scripts for expressing pain. More importantly, pain poses a threat to societal normality, triggering a defensive response from the social environment that pressures the sufferer to quickly restore balance by resuming their roles.

The findings also show how the belief in the interconnectedness of somatic, psychological, and emotional aspects of pain may contribute to justifying these pressures. This belief allows individuals to rationalize the expectation for sufferers to recover and return to normal life, reinforcing the individualization of pain. Thus, while public discourse may outwardly express empathy, practical behaviors often contradict these sentiments, with individuals placing responsibility on sufferers to manage and overcome their pain.

Ultimately, raising awareness and empathy around pain is not sufficient. This point cannot be overemphasized, especially with regard to healthcare professionals and other expert systems involved in pain management, as our results suggest that many continue to have a systemic attitude of mistrust towards the afflicted. This, however, is not enough. A sympathetic and empathetic narrative around the pain of others seems to have positively permeated society but, ultimately, this does not necessarily translate into more compassionate behavior towards those who suffer. Rather, in the face of the pain of others, the tendency seems to be to preserve one’s own normality.

Therefore, it seems like efforts should focus on addressing the systemic pressures and organizational structures that make individuals responsible for both their own pain and the pain of others in order to relieve the everyday burdens faced by those suffering. We believe that this is the main contribution of our study to pain research, although it is in line with other recent studies in the field [37]. There is still much to explore and reflect on in this direction, particularly in its practical implications for pain management, as we hope to do in future research.

## Figures and Tables

**Table 1:** Sample of groups.

Group	Composition	Place	Date	Duration
G1	Women aged 51–60	Seville	15 January 2019	150 min
G2	Women aged 65 and above	Seville	16 January 2019	116 min
G3	Men aged 31–50, private sector service workers.	Seville	16 January 2019	140 min
G4	Women aged 31–50, unskilled workers.	Cordoba	23 January 2019	110 min
G5	Men and women aged 31–50, public servants.	Saragossa	28 January 2019	150 min
G6	Men and women aged 18–30, unemployed and students.	Madrid	30 January 2019	135 min
G7	Men and women aged 31–65, entrepreneurs and self-employed.	Madrid	31 January 2019	140 min
G8	Men aged 51–65, farmers, fishermen, and construction workers.	Conil	10 February 2019	100 min

Source: Authors’ own elaboration.

**Table 2:** Interview sample.

Type of Pain	Pain	Duration	Interview
Physical Pain	Arthrosis and osteoporosis	11 min	I1
	Migraine	47 min	I2
	Obesity	20 min	I3
	Fibromyalgia	76 min	I4
	Lupus	32 min	I5
	Myalgic encephalomyelitis	41 min	I6
Psychological pain	Depressive schizoaffective disorder	88 min	I7
	Borderline personality disorder	76 min	I8
	Chronic depression	80 min	I9
	Gambling addiction	5 min	I10
Emotional pain	Death of a daughter	87 min	I11
	Death of a spouse	78 min	I12
	Relationship breakdown	54 min	I13
	Rejection due to sexual orientation	70 min	I14
Social pain	Chronic fatigue syndrome	42 min	I15
	MCS/ES comorbidities	103 min	I16
	Gender-based violence	74 min	I17
	Bullying	69 min	I18
	Adoption	114 min	I19

Source: Authors’ own elaboration.

## Data Availability

Data are unavailable due to privacy or ethical restrictions. The complete transcripts of all of the interviews carried out in the investigation and described in the material and methods section cannot be made public in any type of repository, because they could identify the people interviewed in breach of the anonymity and privacy commitment that we have with them.
